# Prediction model of ocular metastasis from primary liver cancer: Machine learning‐based development and interpretation study

**DOI:** 10.1002/cam4.6540

**Published:** 2023-10-05

**Authors:** Jin‐Qi Sun, Shi‐Nan Wu, Zheng‐Lin Mou, Jia‐Yi Wen, Hong Wei, Jie Zou, Qing‐Jian Li, Zhao‐Lin Liu, San Hua Xu, Min Kang, Qian Ling, Hui Huang, Xu Chen, Yi‐Xin Wang, Xu‐Lin Liao, Gang Tan, Yi Shao

**Affiliations:** ^1^ Fuxing Hospital, The Eighth Clinical Medical College Capital Medical University Beijing People's Republic of China; ^2^ Department of Ophthalmology The First Affiliated Hospital of Nanchang University, Jiangxi Branch of the National Clinical Research Center for Ocular Disease Nanchang People's Republic of China; ^3^ Fujian Provincial Key Laboratory of Ophthalmology and Visual Science, Eye Institute of Xiamen University School of Medicine, Xiamen University Xiamen People's Republic of China; ^4^ Department of Ophthalmology The First Affiliated Hospital of University of South China, Hunan Branch of The National Clinical Research Center for Ocular Disease Hengyang People's Republic of China; ^5^ Department of Ophthalmology and Visual Sciences Maastricht University Maastricht Netherlands; ^6^ School of Optometry and Vision Sciences Cardiff University Cardiff UK; ^7^ Department of Ophthalmology and Visual Sciences The Chinese University of Hong Kong Hong Kong People's Republic of China

**Keywords:** machine learning, ocular metastasis, primary liver cancer, Shapley additive explanations, XGBoost

## Abstract

**Background:**

Ocular metastasis (OM) is a rare metastatic site of primary liver cancer (PLC). The purpose of this study was to establish a clinical predictive model of OM in PLC patients based on machine learning (ML).

**Methods:**

We retrospectively collected the clinical data of 1540 PLC patients and divided it into a training set and an internal test set in a 7:3 proportion. PLC patients were divided into OM and non‐ocular metastasis (NOM) groups, and univariate logistic regression analysis was performed between the two groups. The variables with univariate logistic analysis *p* < 0.05 were selected for the ML model. We constructed six ML models, which were internally verified by 10‐fold cross‐validation. The prediction performance of each ML model was evaluated by receiver operating characteristic curves (ROCs). We also constructed a web calculator based on the optimal performance ML model to personalize the risk probability for OM.

**Results:**

Six variables were selected for the ML model. The extreme gradient boost (XGB) ML model achieved the optimal differential diagnosis ability, with an area under the curve (AUC) = 0.993, accuracy = 0.992, sensitivity = 0.998, and specificity = 0.984. Based on these results, an online web calculator was constructed by using the XGB ML model to help clinicians diagnose and treat the risk probability of OM in PLC patients. Finally, the Shapley additive explanations (SHAP) library was used to obtain the six most important risk factors for OM in PLC patients: CA125, ALP, AFP, TG, CA199, and CEA.

**Conclusion:**

We used the XGB model to establish a risk prediction model of OM in PLC patients. The predictive model can help identify PLC patients with a high risk of OM, provide early and personalized diagnosis and treatment, reduce the poor prognosis of OM patients, and improve the quality of life of PLC patients.

## 1 INTRODUCTION

**FIGURE 1:**
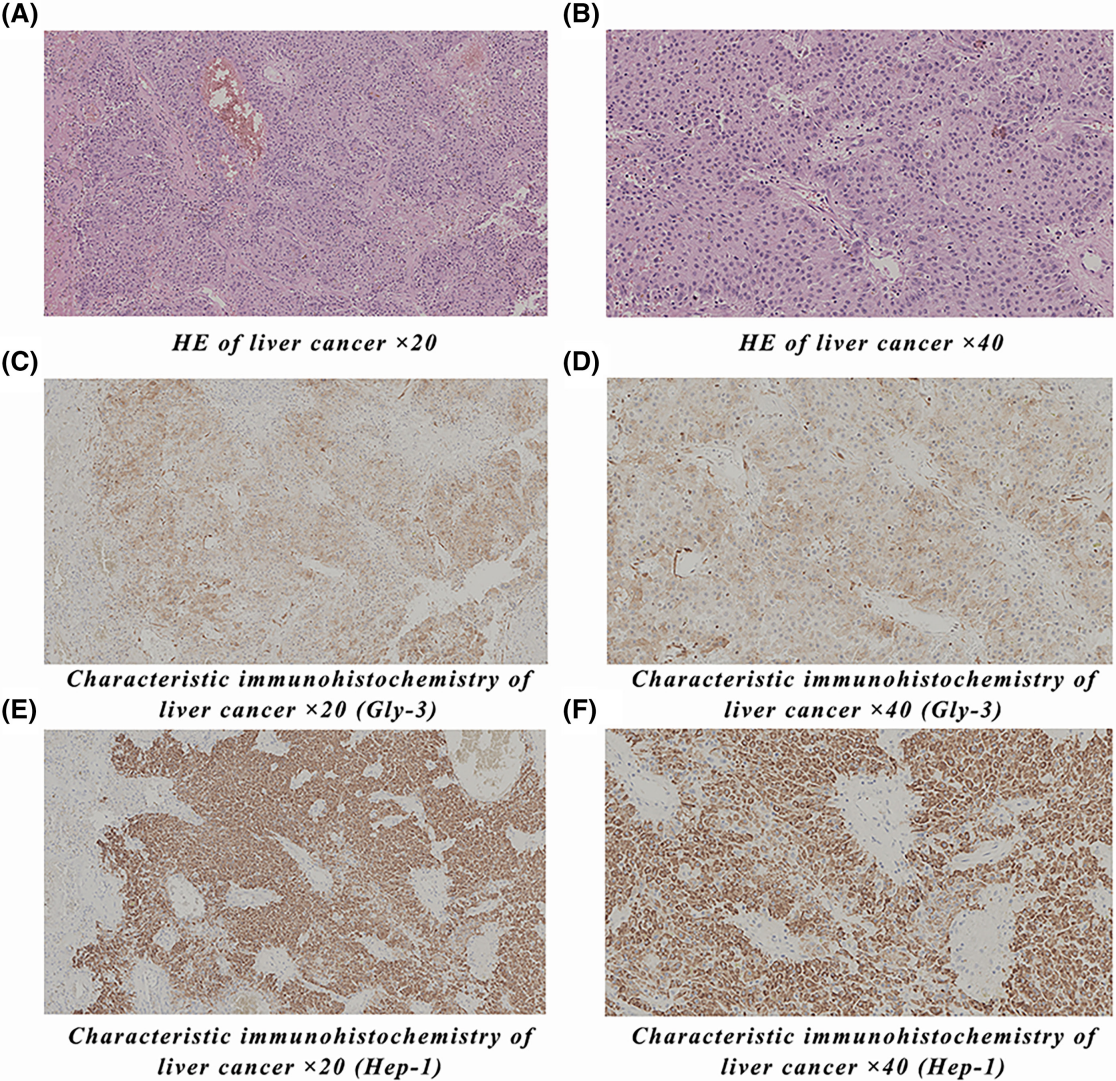
HE and characteristic immunohistochemistry of liver cancer. (A, B) are HE‐stained section images of patients with liver cancer, (C, D) are Gly‐3 immunohistochemical staining images of patients with liver cancer, and (E, F) are Hep‐1 immunohistochemical staining images of patients with liver cancer. HE, Hematoxylin eosin staining; Glypican‐3, Gly‐3.

Primary liver cancer (PLC) is a common malignant tumor of the liver and the sixth most common cancer in the world. PLC mainly includes hepatocellular carcinoma (HCC), intrahepatic cholangiocarcinoma (ICC), and mixed liver cancer, of which HCC accounts for 80%–90% and ICC accounts for 10%–15%.^1^ In the process of microscopic examination of the tissue sections of patients with liver cancer, the common immunohistochemical markers of liver cancer patients were Gly‐3 and Hep‐1, and they were observed by HE staining sections (Figure 1). Distant metastasis is the main cause of death in PLC patients and often involves the lungs, abdomen, mediastinal lymph nodes, and even the brain.^2^ Ocular metastasis (OM) is rare due to the presence of the blood‐eye barrier and fewer lymphatic vessels in the eye. Once OM occurs in PLC patients, it often indicates that their prognosis is very poor, and most of the patients with OM have metastasis in other sites. Therefore, early diagnosis and prediction of distant metastasis and the risk of OM are particularly important for the prognosis of PLC patients. Tumor markers are active components produced by the metabolism and secretion of tumor cells that can accurately reflect tumor properties and be used as serological indicators for auxiliary diagnosis of all types of tumors. Tumor markers can be significantly increased in the blood of PLC patients and can be used as prognostic indicators, which is conducive to the early diagnosis and prognostic evaluation of liver cancer; however, their sensitivity and specificity in the diagnosis of distant metastasis in liver cancer remain low.^3^ Our previous retrospective analysis of PLC patients with hypertension showed that the area under the curve (AUC), sensitivity, and specificity of AFP combined with CA125 in the differential diagnosis of the OM group and non‐ocular metastasis (NOM) group were 0.875, 0.762, and 0.884, respectively.^4^ However, the real clinical application value is limited, and the accuracy and various evaluation indicators still have a large rate of missed diagnosis and misdiagnosis. With the development of diagnostic and screening technology in recent years, the diagnostic rate of PLC with extrahepatic metastasis is increasing. Currently, there is no standard treatment for PLC patients with distant metastasis. The existing guidelines recommend targeted therapy, systemic chemotherapy, or the best supportive care for PLC patients with distant metastasis, but the therapeutic effect is limited.^5^ Therefore, most of the research work has been committed to finding effective prediction methods, prognosis assessment, and timely intervention.

With the rise of big data and artificial intelligence in recent years, more machine learning (ML) and deep learning methods have been applied to clinical research, and compared with traditional prediction models, ML models often have higher accuracy and sensitivity based on big data. We had previously developed an ML model for bone metastasis in breast cancer patients based on the SEER database and hospital data, in which the AUC of the extreme gradient boosting (XGB) ML model for predicting bone metastasis in breast cancer patients could reach 0.888, and the accuracy could reach 0.803.^6^ In addition, we have also studied the automated classification and identification of three different types of meibomian gland dysfunction in ophthalmology based on the deep learning DenseNet‐169 neural network, with a sensitivity of 0.88 and specificity greater than 0.95.^7^ Yamashita et al also developed an HCC‐SucvNet model based on deep learning survival analysis to predict recurrence after PLC hepatectomy, with an AUC of 0.724 in the internal verification set and 0.683 in the external test set, and provided the risk score for recurrence in each patient.^8^ However, currently there are few studies on the ML model of distant metastasis in PLC patients, especially for OM. Therefore, the purpose of this study was to further improve the accuracy of predicting the risk of OM in PLC patients, construct several prediction models based on biomarkers to quantify the risk of OM, compare the performance of different ML models with the optimized traditional clinical prediction model, explain the different effects of serological indexes and tumor markers on OM in PLC patients, and choose the best ML model. A web calculator was developed to determine a personalized prediction of OM in PLC patients to improve their prognosis. The significance of our study is to build a clinical prediction model of early OM in PLC patients based on the ML model, providing a mathematical model for early assessment of the clinical prognosis of PLC patients and assisting the clinical treatment of PLC patients.

## 2 METHODS

### 2.1 Subjects

**FIGURE 2:**
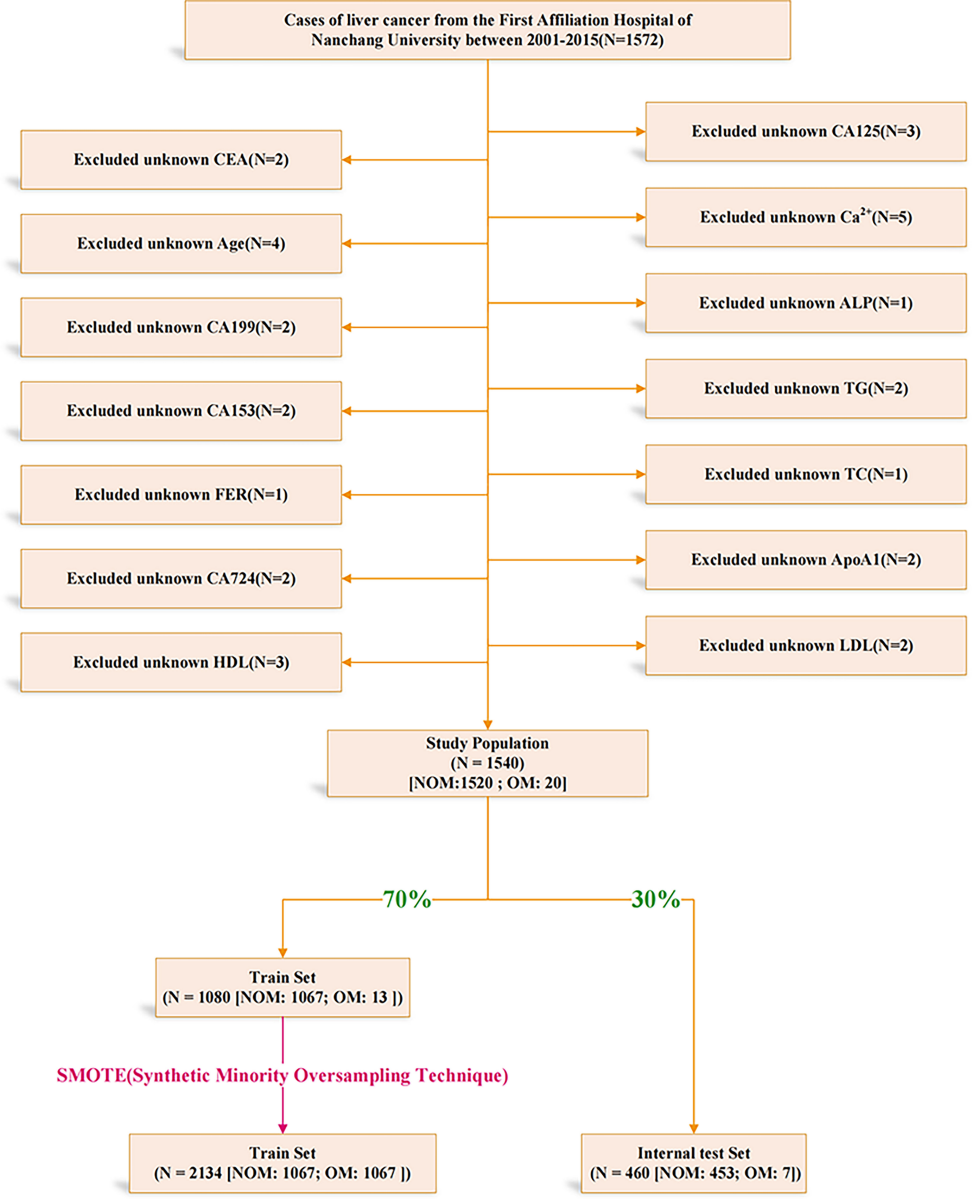
The flow chart of data cleaning. We cleaned the data from 2001 to 2015 and eventually included 1540 PLC cases, 20 of which had ocular metastases. Divide the training set and the test set according to a ratio of 7:3. We put SMOTE method on the training set to deal with the unbalanced data and did not process the test set to preserve the original ratio.

The population data were collected from the First Affiliated Hospital of Nanchang University. We retrospectively collected the clinical data of 1572 patients with liver cancer from August 2001 to May 2015 and screened for missing data in each patient. Finally, we included 1540 PLC patients in this study, including 1520 NOM patients and 20 OM patients. The inclusion criteria for the OM group were eye metastases in PLC patients diagnosed by CT and MRI and confirmed by histology and cytology. For the specific screening process, please see the flow chart in Figure 2. The inclusion criteria of PLC patients were the following: (1) cancerous liver tissue (confirmed by histopathological biopsy); (2) no contraindication for magnetic resonance imaging (MRI); and (3) nonmetastatic liver cancer. The exclusion criteria of the OM group were the following: (1) primary malignant tumor of the eye; and (2) benign tumor of the eye. The experimental design of this study was explained to the patients and written informed consent was provided by all study participants. This study was performed in accordance with the tenets of the Declaration of Helsinki and was approved by the Medical Ethics Committee of the First Affiliated Hospital of Nanchang University and the approved number was cdyfy20170411.

### 2.2 Data collection

Medical records of all participants were collected to acquire basic information about their age, sex, and pathological tumor type. In addition, serological data were collected from both groups of subjects, including hemoglobin (Hb), serum calcium, lipoprotein a, apolipoprotein B (Apo B), apolipoprotein A1 (Apo A1), low‐density lipoprotein (LDL), high‐density lipoprotein (HDL), triglycerides (TG), total cholesterol (TC), alkaline phosphatase (ALP), ferritin (FER), carbohydrate antigen‐724 (CA724), CA153, CA199, CA125, carcinoembryonic antigen (CEA), and alpha‐fetoprotein (AFP).

### 2.3 Statistical analysis

The statistical analysis was carried out by Python (version 3.8) and R software (version 4.0.2). The synthetic minority oversampling (synthetic minority oversampling technique; SMOTE) technique is applied to the original dataset using Python to reduce the impact of unbalanced data on machine learning partitioning datasets and subsequent verification.^9^ Using the method of stratified random sampling, the data set after SMOTE is randomly divided into the training set and internal test set according to the proportion of 7:3. Use the training set to build the ML model, and use the internal test set to verify and evaluate the model. The Mann–Whitney *U*‐test is used for continuous non‐normal distribution data, and the chi‐squared test is used for classified counting data. In univariate analysis, the variables with *p* < 0.05 were included in the construction of the ML model, and multivariate logistic regression (LR) was used to determine the risk factors of OM in PLC patients. The Python programming language (version 3.8) was also used to develop and evaluate ML models and design web calculators. For model interpretation, the SHAP was implemented using the Python SHAP package. *p* < 0.05 was considered statistically significant.

### 2.4 Data preprocessing and feature engineering

The label coding method was used to deal with the classification variables, such as gender, stage, pathological type, and AFP comparison. Univariate analysis was used to select meaningful feature combinations to predict the risk of OM in PLC patients. We used the SHAP package to establish the order of importance of risk factors of OM in PLC patients. SHAP is a method based on cooperative game theory to interpret the results of the prediction model. This method can quantify the SHAP value of each characteristic variable, which represents the contribution of different characteristics to the predictive risk of OM in PLC patients. For each sample, the model can produce a predictive value, and the sum or average value of the absolute value Shapley of each feature of all samples is the overall importance score of the feature. In addition, the SHAP method also proves that each eigenvalue has a positive or negative effect on the prediction results, which is similar to the coefficient value in logical regression. When the SHAP value is positive, it indicates that the corresponding feature leads to a higher probability of OM risk, while when the SHAP value is negative, it indicates that the corresponding feature leads to a lower risk of OM.^10^, ^11^

### 2.5 Machine learning model building

All the algorithm models are based on scikit‐learn (version 1.1.1). In this study, we use six different ML models: multilayer perceptron (MLP) model^12^, ^13^; AdaBoost (AB) model^14^; Bagging (BAG) classification model^15^; LR model^16^; gradient boosting machine (GBM) model^17^; XGB model. The ML algorithm was trained and adjusted to predict OM in PLC patients. The random search method in scikit‐learn is used to adjust the super parameters of the different models. Then, through the internal 10‐fold cross‐validation of the whole data, the predictive performance of the ML model was evaluated. Then we chose the model with the optimal performance to construct a web calculator. Model parameter settings are detailed in Data S1. The parameter settings link is accessible from https://github.com/Wu‐Shi‐Nan/liver_cancer_ml_parameters/blob/main/model_parameter_settings.txt.

## 3 RESULTS

### 3.1 Demographic features

There were significant differences in tumor staging, AFP, CA125, CA199, and ALP between the NOM and OM groups (*p < 0.05*). The proportion of Stage IV patients and the contents of AFP, CA125, CA199, and ALP in the OM group were significantly higher than in the NOM group. Other related indicators can be found in Table 1.

**TABLE 1:** Comparison of baseline data between the two groups.

Variables	Total (*n* = 1540)	NOM group(*n* = 1520)	OM group (*n* = 20)	*p value*
Gender, *n* (%)				
Male	1320 (86)	1304 (86)	16 (80)	0.52
Female	220 (14)	216 (14)	4 (20)	
Stage, *n* (%)				
Stage 1	1 (0)	1 (0)	0 (0)	0.002*
Stage 2	60 (4)	60 (4)	0 (0)	
Stage 3	293 (19)	293 (19)	0 (0)	
Stage 4	97 (6)	92 (6)	5 (25)	
Unknown	1089 (71)	1074 (71)	15 (75)	
Pathological type, *n* (%)				
Hepatocellular carcinoma	286 (19)	286 (19)	0 (0)	0.07
Cholangiocarcinoma	42 (3)	42 (3)	0 (0)	
Mixed hepatocellular carcinoma	1 (0)	1 (0)	0 (0)	
Unknown	1211 (79)	1191 (78)	20 (100)	
AFP, *n* (%)				
<400	765 (50)	764 (50)	1 (5)	<0.001*
≥400	775 (50)	756 (50)	19 (95)	
Age, Median (Q1, Q3)	51 (42, 61)	52 (42, 61)	49 (44.75, 52.5)	0.174
CEA, Median (Q1, Q3)	2.15 (1.22, 3.62)	2.17 (1.22, 3.6)	2.06 (1, 7.52)	0.954
CA125, Median (Q1, Q3)	34 (13.75, 143)	33 (13.66, 138.68)	449.6 (198.58, 675.43)	<0.001*
CA199, Median (Q1, Q3)	24.04 (12.3, 56.49)	24 (12.27, 55.95)	46.86 (19.11, 189.45)	0.028*
CA153, Median (Q1, Q3)	14.63 (10.94, 17.06)	14.63 (10.94, 17.07)	13.82 (12.24, 14.93)	0.854
CA724, Median (Q1, Q3)	5 (2.92, 9.7)	5 (2.92, 9.7)	4.08 (2.95, 8.99)	0.959
FER, Median (Q1, Q3)	214 (133, 321)	214 (133, 321)	204.5 (119.03, 217)	0.162
ALP, Median (Q1, Q3)	117 (79, 183)	116 (79, 182)	198.5 (131, 298.25)	<0.001*
TC, Median (Q1, Q3)	4.19 (3.04, 5.12)	4.19 (3.04, 5.11)	3.92 (3.44, 5.31)	0.576
TG, Median (Q1, Q3)	1.02 (0.79, 1.54)	1.02 (0.79, 1.54)	1.36 (0.76, 2.08)	0.189
HDL, Median (Q1, Q3)	1.23 (0.99, 1.54)	1.23 (1, 1.54)	1.27 (0.93, 1.46)	0.904
LDL, Median (Q1, Q3)	2.33 (1.57, 3.11)	2.33 (1.57, 3.11)	2.44 (1.62, 3.66)	0.505
Apo A1, Median (Q1, Q3)	1.43 (1.3, 1.8)	1.43 (1.3, 1.8)	1.42 (1.34, 1.96)	0.271
Apo B, Median (Q1, Q3)	0.89 (0.61, 1.16)	0.89 (0.61, 1.16)	0.95 (0.67, 1.17)	0.641
Lipoprotein a, Median (Q1, Q3)	132 (54, 332)	137 (54, 332)	78 (53, 148)	0.163
Ca^2+^, Median (Q1, Q3)	2.13 (2, 2.27)	2.13 (2, 2.27)	2.11 (2.02, 2.18)	0.768
Hb, Median (Q1, Q3)	119 (103, 134)	119 (103, 134)	122.5 (112.5, 133)	0.474

Abbreviations: ALP, alkaline phosphatase; Apo B, apolipoprotein B; ApoA1, apolipoprotein A1; FER, ferritin; Hb, hemoglobin; HDL, high‐density lipoprotein; LDL, low‐density lipoprotein; TC, total cholesterol; TG, triglycerides.

* *p < 0.05* represented statistically significant.

### 3.2 Univariate and multivariate LR analysis

**FIGURE 3:**
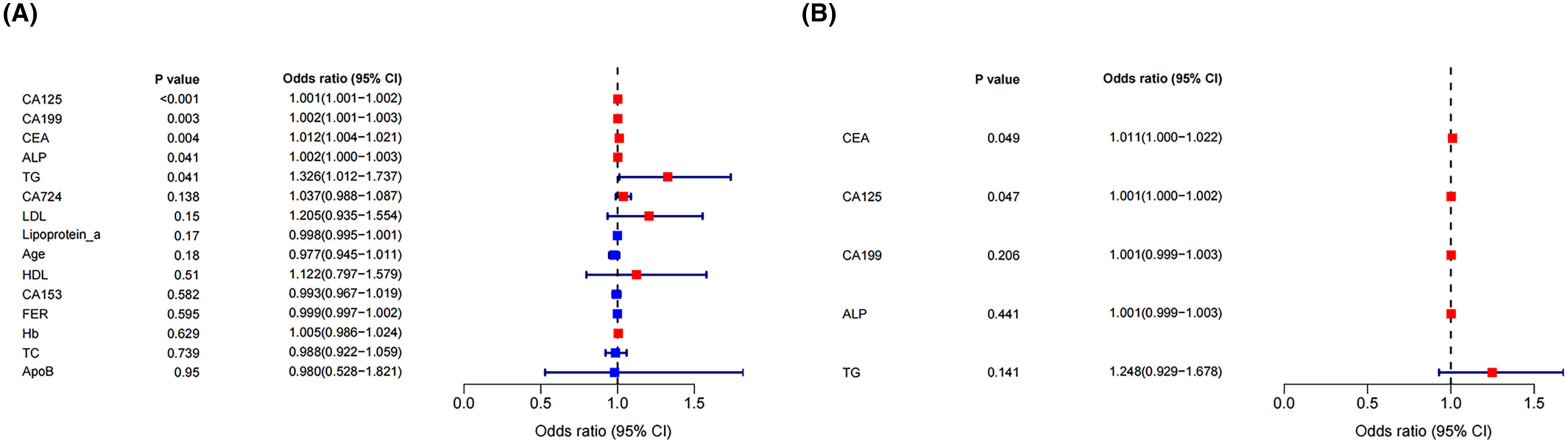
Univariate and multivariate logistic regression (LR) results in forest map. (A) Showed the univariate LR results in the forest map and (B) showed the multivariate LR results in the forest map. ALP, alkaline phosphatase; Apo A1, apolipoprotein A1; Apo B, apolipoprotein B; CI, confidence interval; FER, ferritin; Hb, hemoglobin; HDL, high‐density lipoprotein; LDL, low‐density lipoprotein; OR, odds ratio; TC, total cholesterol; TG, triglycerides.

Through the establishment of the univariate LR model, we selected the features with a *p* < 0.05 from the univariate LR analysis and then conducted a multivariate LR analysis to determine the risk factors for OM in PLC patients. In univariate LR, AFP, CEA, CA125, CA199, ALP, and TG were risk factors for OM, and these indexes were incorporated into the characteristic variables of six ML models. Multivariate LR showed that AFP, CA125, and CA199 were independent risk factors for OM in PLC patients (*p < 0.05*). If the odds ratio (OR) and 95% confidence interval (CI) of these risk factors are greater than one, then they are high‐risk predictors of OM (Figure 3; Table 2).

**TABLE 2:** Univariate and multivariate logistic regression.

Characteristics	Category	Univariate analysis		Multivariate analysis	
		OR (95% CI)	*p‐*Value	OR (95% CI)	*p‐*Value
Gender	Male	Ref	Ref	Ref	Ref
	Female	1.516 (0.502–4.579)	0.46	\	\
Stage	Stage 1	Ref	Ref	Ref	Ref
	Stage 2	1 (0‐Inf)	1	\	\
	Stage 3	1 (0‐Inf)	1	\	\
	Stage 4	343163599.287 (0‐Inf)	1	\	\
Pathological type	Hepatocellular carcinoma (HCC)	Ref	Ref	Ref	Ref
	Cholangiocarcinoma	1 (0‐Inf)	1	\	\
	Mixed hepatocellular carcinoma	1 (0‐Inf)	1	\	\
	Unknown	14349881.235 (0‐Inf)	0.987	\	\
AFP	<400	Ref	Ref	Ref	Ref
	≥400	19.201 (2.564–143.792)	0.004*	26.813 (3.142–228.838)	0.003*
CEA	\	1.012 (1.004–1.021)	0.004*	1.011 (1–1.022)	0.049*
CA125	\	1.001 (1.001–1.002)	<0.001*	1.001 (1–1.002)	0.047*
CA199	\	1.002 (1.001–1.003)	0.003*	1.001 (0.999–1.003)	0.206
ALP	\	1.002 (1–1.003)	0.041*	1.001 (0.999–1.003)	0.441
TG	\	1.326 (1.012–1.737)	0.041*	1.248 (0.929–1.678)	0.141
Age	\	0.977 (0.945–1.011)	0.18	\	\
CA724	\	1.037 (0.988–1.087)	0.138	\	\
FER	\	0.999 (0.997–1.002)	0.595	\	\
LDL	\	1.205 (0.935–1.554)	0.15	\	\
CA153	\	0.993 (0.967–1.019)	0.582	\	\
Apo B	\	0.98 (0.528–1.821)	0.95	\	\
HDL	\	1.122 (0.797–1.579)	0.51	\	\
Ca	\	0.902 (0.173–4.695)	0.902	\	\
TC	\	0.988 (0.922–1.059)	0.739	\	\
Lipoprotein a	\	0.998 (0.995–1.001)	0.17	\	\
Apo A1	\	1.706 (0.674–4.317)	0.26	\	\
Hb	\	1.005 (0.986–1.024)	0.629	\	\

Abbreviations: ALP, alkaline phosphatase; Apo B, apolipoprotein B; ApoA1, apolipoprotein A1; CI, confidence interval; FER, ferritin; Hb, hemoglobin; HDL, high‐density lipoprotein; LDL, low‐density lipoprotein; OR, odds ratio; TC, total cholesterol; TG, triglycerides.

* *p < 0.05* represented statistically significant.

### 3.3 Machine learning model performance

**FIGURE 4:**
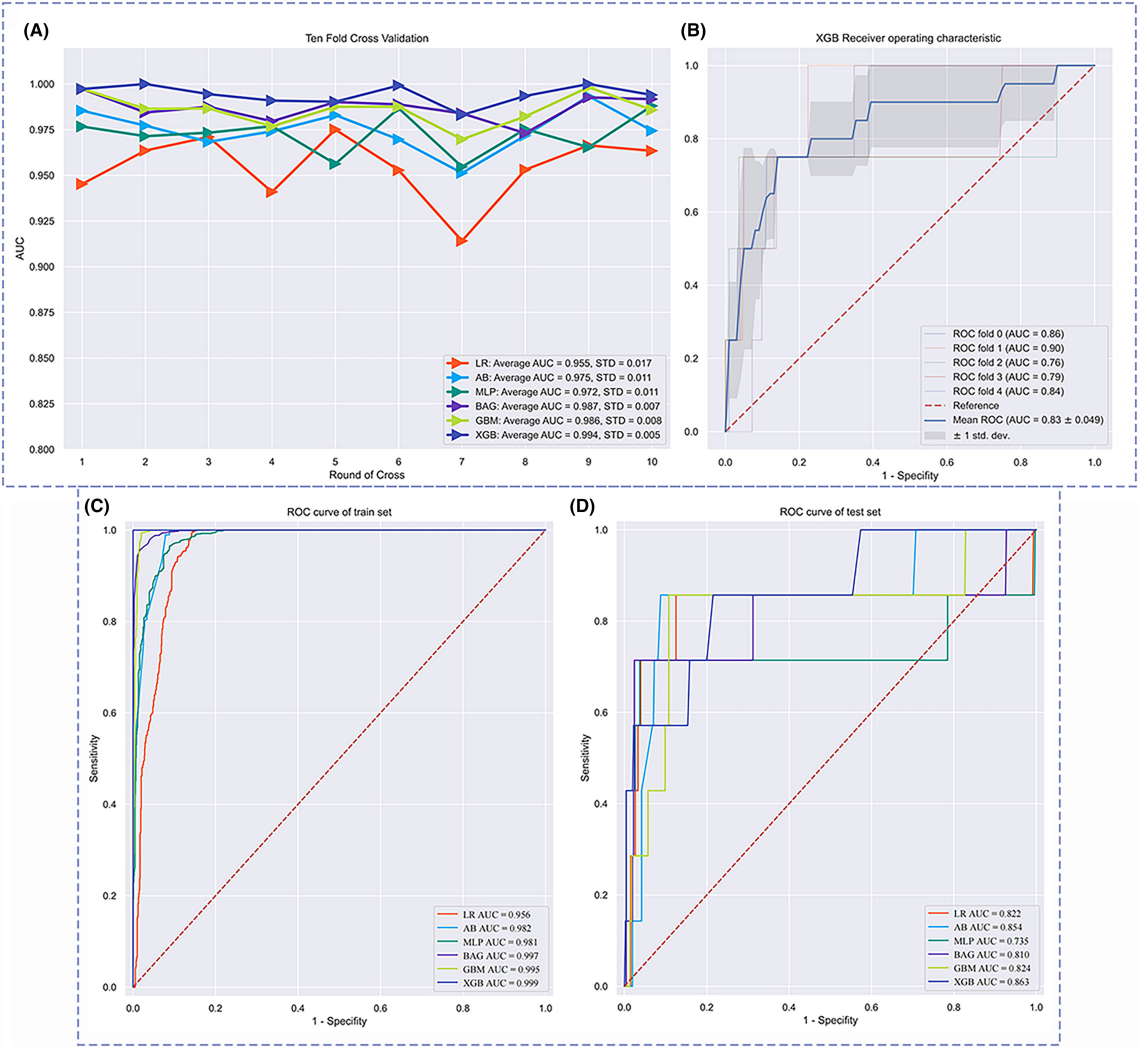
Validation of machine learning algorithms. **(**A) AUC values of 10‐fold cross‐validation. (B) Five cross‐validation results for the best machine learning model of XGB. (C) receiver operating characteristic curve of all machine learning methods in the train set. (D) Receiver operating characteristic curve of all machine learning methods in the test set. AUC is used as an indicator of performance, the XGB model achieved the best predictive performance, and the MLP model had the lowest predictive performance. AB, adaptive boosting; AUC, area under the curve; BAG, bootstrapped aggregating; GBM, gradient boosting machine; LR, logistic regression; ML, machine learning; MLP, multilayer perceptron; ROC, receiver operating characteristic; XGB, extreme gradient boost.

**FIGURE 5:**
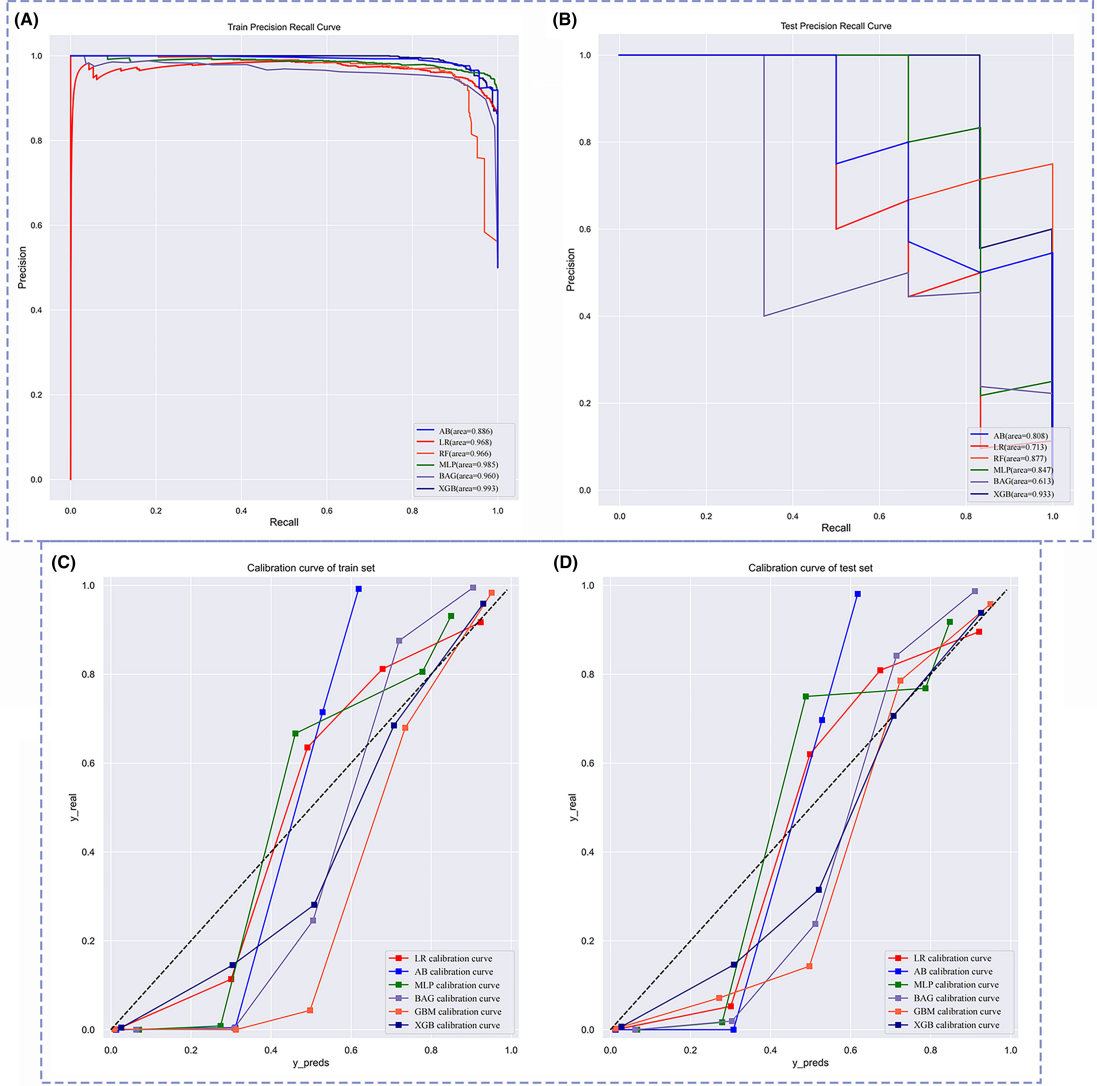
Precision recall curve and a calibration curve of each machine learning model. **(**A, B) showed the precision‐recall curve of the train set and test set; (C, D)showed the calibration curve of the train set and test set. In the six machine models, the XGB model showed the best performance in test set data.

**FIGURE 6:**
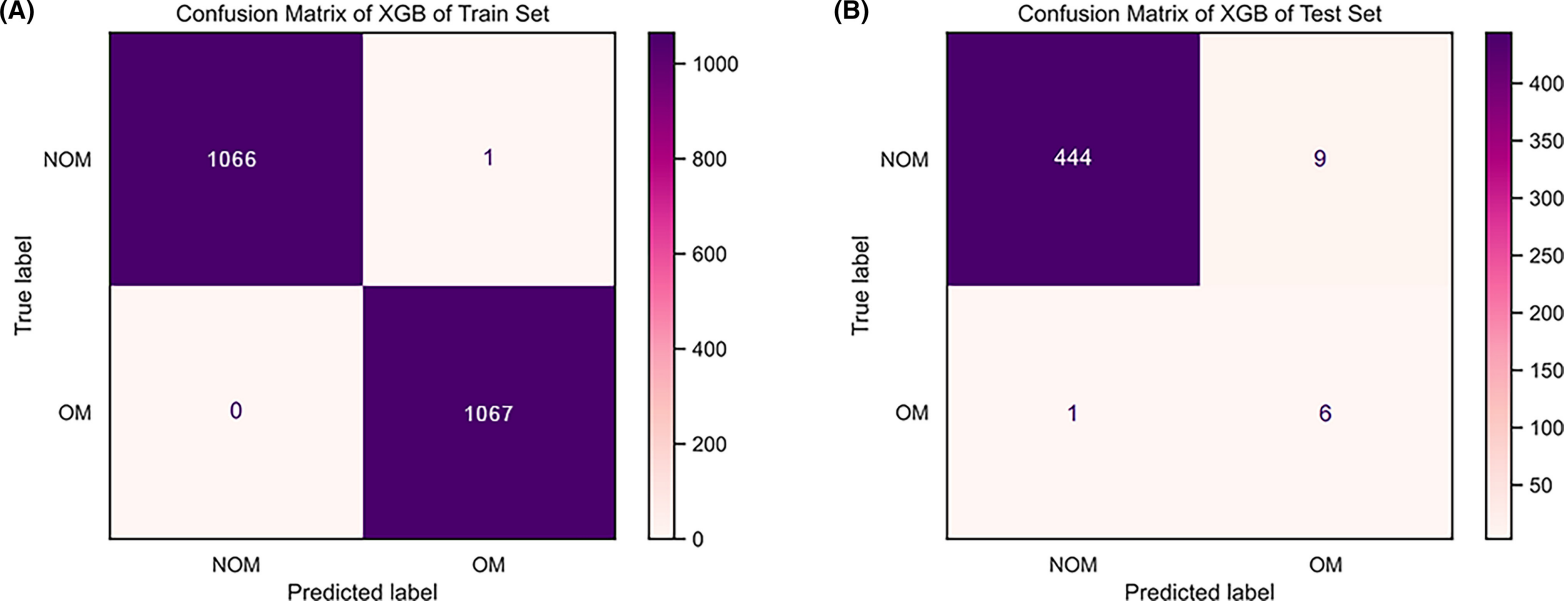
Confusion matrix of six machine learning models. The best correct classification (accuracy) of metastatic ocular in the test set for the XGB model was 0.978. AB, adaptive boosting; BAG, bootstrapped aggregating; GBM, gradient boosting machine; LR, logistic regression; MLP, multilayer perceptron; NOM, none‐ocular metastatic; OM, ocular metastatic; XGB, extreme gradient boost.

**FIGURE 7:**
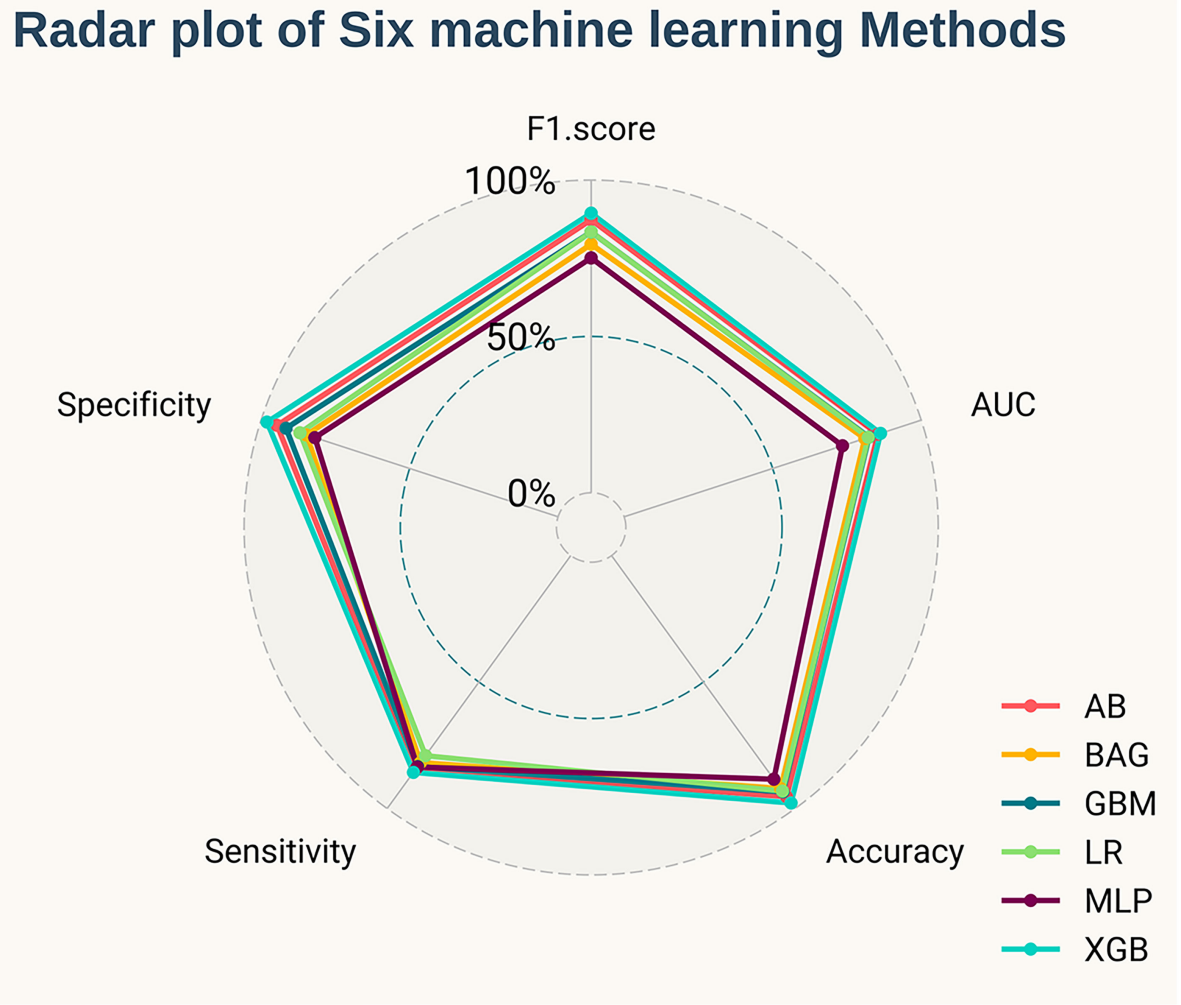
Radar plot of six machine learning methods. Among the six machine learning models, XGB showed the best performance in F1 score, AUC, accuracy, sensitivity, and specificity. AB, adaptive boosting; AUC, the area under the ROC curve; BAG, bootstrapped aggregating; GBM, gradient boosting machine; LR, logistic regression; MLP, multilayer perceptron; XGB, extreme gradient boost.

We used six different ML models, namely MLP, AB, BAG, LR, GBM, and XGB, to evaluate the risk probability and related accuracy of OM in PLC patients. The prediction performance of all models was evaluated with 10‐fold cross‐validation of training sets and fivefold cross‐validation based on the optimal ML model, as shown in Figure 4A,B. The results from the training sets showed that the XGB model performed best, with an F1 score = 0.894, accuracy = 0.978, sensitivity = 0.857, and specificity =0.980 (Table 3). The results of 10‐fold cross‐validation showed that the AUC of XGB = 0.994 and the standard error = 0.005, which was better than other ML models and the traditional LR model. We built a fivefold cross‐validation based on the optimal ML model, XGB, in the raw train set to evaluate the stability of the results, where AUC = 0.83 and the standard error = 0.049. ROC curve results for the training set and test set can be seen in Figure 4C,D. AUC value of the XGB model in the training set is 0.999, and the AUC value in the test set is 0.863. According to the processing principle of unbalanced data, we drew the precision‐recall (PR) curve and calibration curve, in which the XGB model performed best. The area under the PR curve in the training set and the test set were 0.993 and 0.933, respectively (Figure 5). The confusion matrix of the results of the XGB ML model was drawn according to the balanced data of the synthetic minority over‐sampling technique (SMOTE) in the train set, as shown in Figure 6A. In the XGB ML algorithm, there were 1066 samples in the NOM group, 1067 in the OM group, and only 1 sample in the wrong prediction. In the test set, there were 444 samples in the NOM group, 6 in the OM group, and 10 samples in the wrong prediction (Figure 6B) (Accuracy = 0.978; Sensitivity = 0.857; Specificity = 0.980). According to the above six ML models, we drew a radar chart to evaluate the maximum values of five indexes. Compared with other ML models, the sensitivity, F1 score, AUC, accuracy, and specificity of XGB had the best values (Figure 7).

**TABLE 3:** Comparison of six machine learning metrics in the test set.

Model	F1 score	AUC	Accuracy	Sensitivity	Specificity
AB	0.873	0.854	0.953	0.847	0.946
LR	0.834	0.822	0.931	0.791	0.869
BAG	0.795	0.81	0.921	0.818	0.847
MLP	0.751	0.735	0.884	0.836	0.819
GBM	0.834	0.824	0.934	0.834	0.916
XGB	0.894	0.863	0.978	0.857	0.980

Abbreviations: AB, adaptive boosting; AUC, the area under the curve; BAG, bootstrapped aggregating; GBM, gradient boosting machine; LR, logistic regression; MLP, multilayer perceptron; XGB, extreme gradient boost.

### 3.4 Importance of feature variables

**FIGURE 8:**
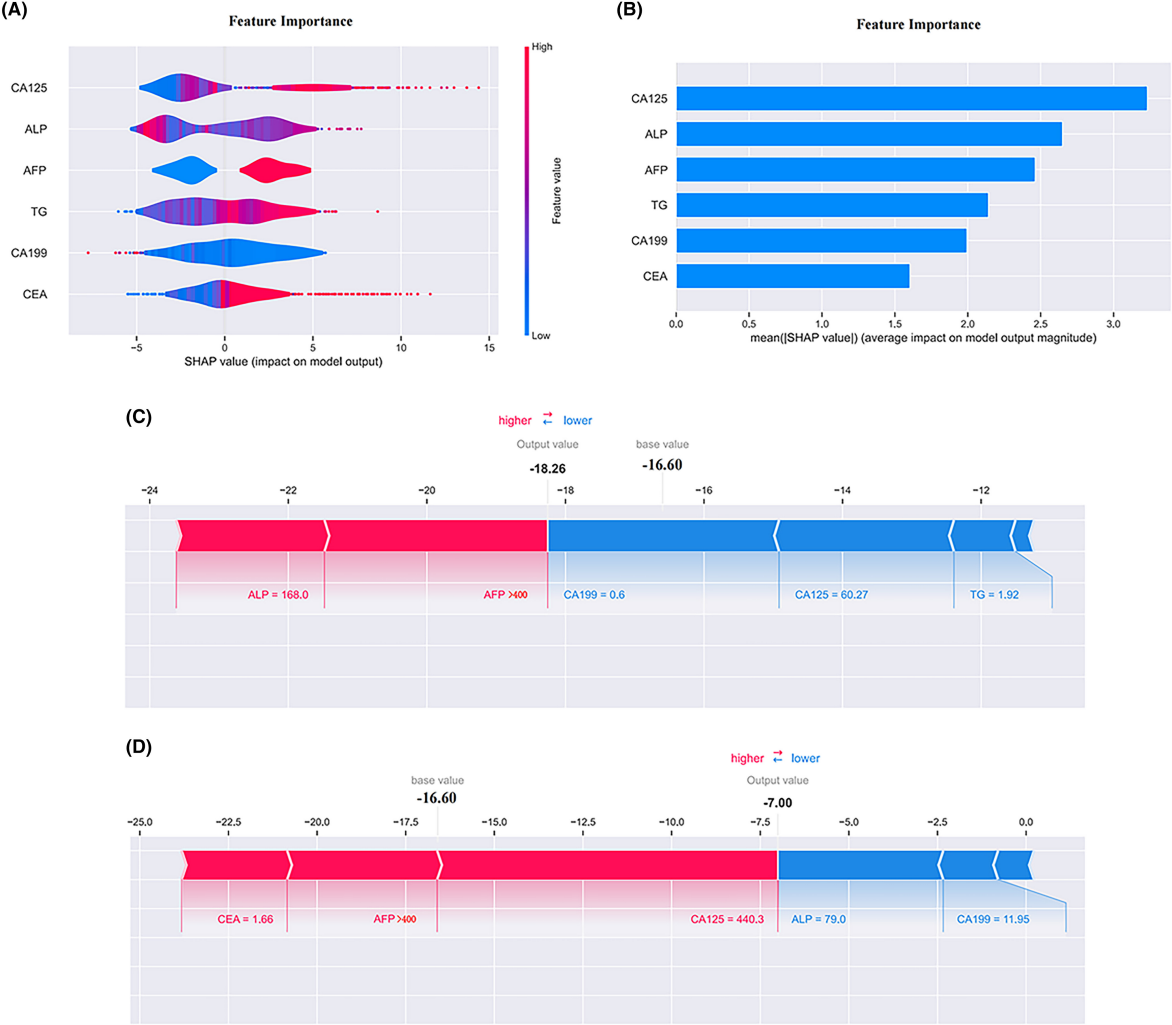
SHAP summary plot and SHAP model explanation of two typical predictions. (A) The features are ranked according to the sum of the SHAP values for all patients, and the SHAP values are used to show the distribution of the effect of each feature on the MLP model outputs. Each dot represents a case in the dataset. The color of a dot indicates the value of the feature, with blue indicating the lowest range and red the highest range. The horizontal axis shows the corresponding SHAP value of the feature. A positive SHAP value contributes to the prediction of rupture and vice versa. (B) Bar chart in descending order of the mean values of importance calculated according to the characteristic variables. (C) showed a low‐risk SHAP interpretation model of ocular metastasis in patients with liver cancer; (D) showed a high‐risk SHAP interpretation model. The base value was −16.60. AFP = 1 represented AFP >400 units. ALP, alkaline phosphatase; SHAP, Shapley additive explanations; TG, triglycerides.

We used the SHAP library to establish a risk factor model of OM in PLC patients based on XGB (Figure 8). The explanation of the risk factor model presented in Figure 8A is as follows: the SHAP values are on the X‐axis, and all values on the left side are the proportion of negative correlations of the predicted value, while the right‐side values are the proportion of positive correlations of the predicted value. The Y‐axis represents the descending order of importance of these features on OM in PLC patients. In the XGB model, the important variables were CA125, ALP, AFP, TG, CA199, and CEA. The specific SHAP value details of each characteristic variable can be seen in Figure 8B. In addition, according to the SHAP library, we selected two subjects, including the OM group and the NOM group. The base value calculated according to our model was −16.6, in which the relative recurrence of the low‐risk group was −18.26 (Figure 8C). The high‐risk factors for OM were ALP = 168 and AFP content >400. Other low‐risk factors were CA199 = 0.6, CA125 = 60.27, and TG = 1.92. The calculated value of relative OM in the high‐risk group was −7 (Figure 8D). In this sample, CEA = 1.66, AFP content >400, and CA125 = 440.3 were the high‐risk factors for OM, while ALP = 79 and CA199 = 11.95 were the low‐risk factors. The samples of the two subjects showed that an AFP content >400 was a common high‐risk factor, while CA199 was a relatively low‐risk factor. For other specific numerical details, please see Figure 8.

### 3.5 Web page calculator

**FIGURE 9:**
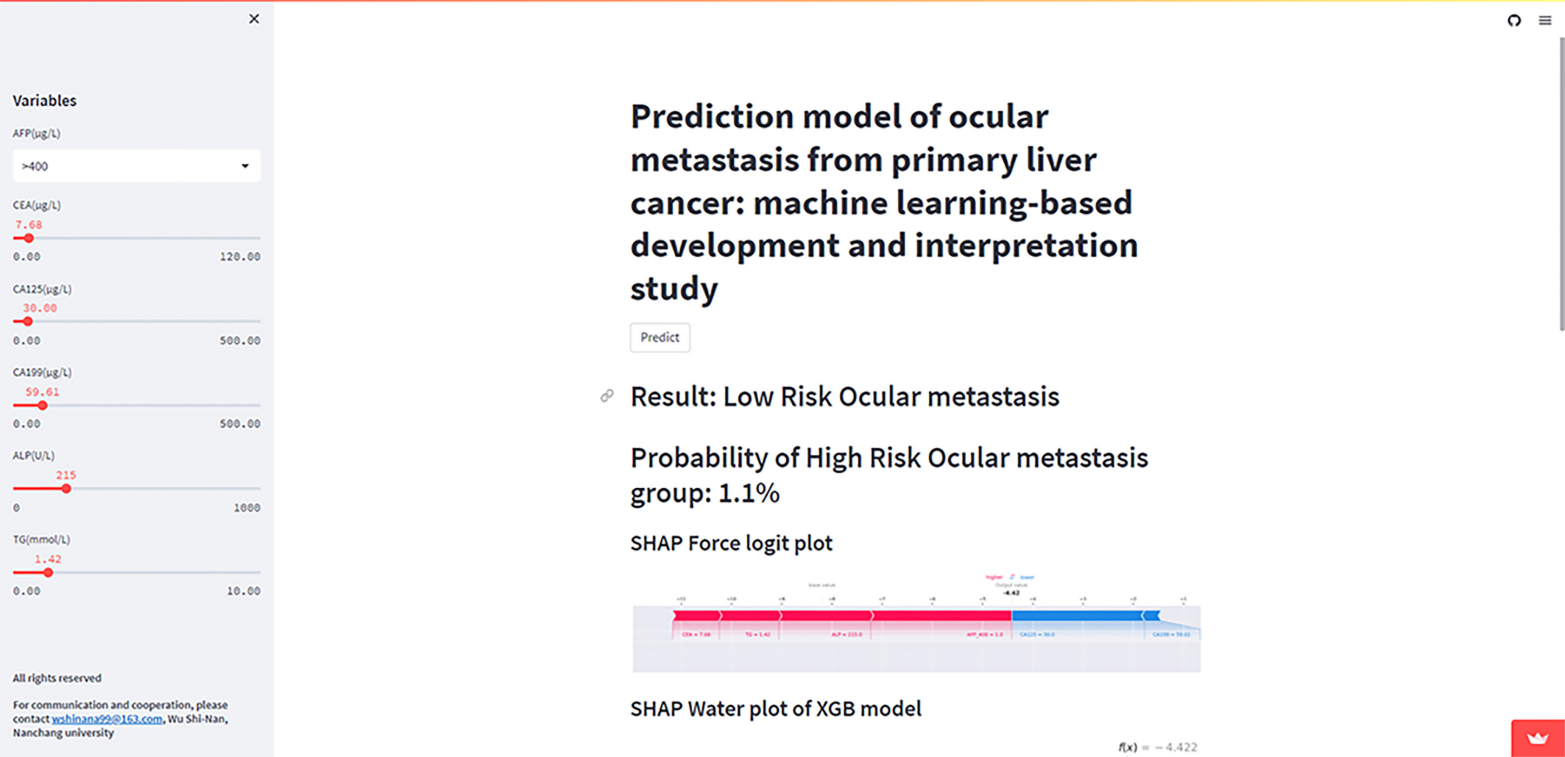
Web calculator for predicting metastatic ocular liver cancer based on extreme gradient boosting model. The URL was https://ml‐cancer‐medicine‐live‐livercancer‐eyemetastis‐zrbnzl.streamlit.app/.

The XGB model had the optimal prediction performance; therefore, the above web predictor was used to predict the risk probability of OM. Users only need to enter the specific number of characteristic variables in the sidebar of the web page and click Predict to obtain the risk probability of OM. In addition, the important factors of eye metastasis were calculated and sorted according to the user variables in real‐time, and the degree of influence of each variable on the results could be intuitively seen.(https://ml‐cancer‐medicine‐live‐livercancer‐eyemetastis‐zrbnzl.streamlit.app/) (Figure 9).

## 4 DISCUSSION

In this study, six ML algorithms were used to predict the risk of OM in PLC patients for the first time, and an XGB model that could be used to predict OM in PLC patients was obtained and explained. The XGB model is based on the concept of gradient tree enhancement for introducing new features to further improve the speed and performance of the classifier and has been widely used in medicine and ecology in recent years.^18^, ^19^ Subsequently, we designed a web calculator based on the XGB model to predict and calculate the probability value of OM to help clinicians make a targeted diagnosis and treatment plan as accurately as possible.

Liver cancer is the second most common cause of cancer‐related death worldwide,^20^ and its incidence has been on the rise in recent years. There are a large number of patients with HBV and HCV in China, which play a vital role in tumorigenesis and are important risk factors for liver cancer.^21^ Epidemiological studies have shown that about 50%–75% of liver cancer patients have distant metastasis in the course of the disease.^22^ The most common sites of metastasis include the lungs and local lymph nodes. The probability of OM from liver cancer is very low; however, once it occurs, the prognosis is very poor. OM is often associated with multiple systemic metastases, and the clinical treatment for its patients is often radiotherapy and chemotherapy.^23^ Thus far, ocular metastases from rectal cancer,^24^ lung cancer,^25^ breast cancer,^26^ gastric cancer,^27^ and esophageal cancer have been reported.^28^ The main clinical manifestations of patients with OM were exophthalmos, pain, ophthalmoplegia, diplopia, and decreased vision.^29^ Our previous studies on OM in patients with liver cancer complicated with hypertension or diabetes showed that AFP and CA125 were independent risk factors for OM.^4^, ^30^ However, these studies did not provide the calculation method for predicting the probability of OM in PLC patients; thus, there remained a gap for real clinical application. In this study, we compared the traditional LR model and other ML models and developed and applied the XGB ML model to the clinical prediction of OM in PLC patients. The XGB model successfully achieved the advantages of high accuracy, sensitivity, and specificity; therefore, we developed a web calculator based on this model to personalize OM prediction in PLC patients. Clinicians only need to input the patient's indicators in the sidebar of the web page to obtain the predicted probability risk of OM.

ML is a mathematical model that applies artificial intelligence under the background of big data and determines the relationship between variables from a large number of data samples. ML has been closely integrated with medicine in recent years and gradually produces the cross direction of the medical industry. It is one of the important branches of data mining.^31^ Thus far, a variety of ML models have been applied to the research of clinical prediction and the combination of computer vision technology and medical imaging. Ding et al collected multicenter data from HCC patients and developed a hybrid ML model based on semantic information combined with XGB to further optimize the treatment decision of early recurrence in these patients.^32^ Xu et al used the ML method to predict bone metastasis in renal cell carcinoma. The comparison of evaluation indexes among various ML methods showed that the XGB ML model had the highest AUC (0.891) in clinical prediction.^33^ In addition, Jiang et al used the XGB model combined with deep learning for early identification of preoperative microvascular invasion in HCC patients, and the differential rate of the AUC could reach 0.906.^34^ In our study, because the probability of OM in PLC patients is low, even when the data of PLC patients were collected for 14 years, the incidence of OM was only 1.3%. To reduce the impact of unbalanced data, we applied the SMOTE method of oversampling. After balancing the data, the AUC in the internal test set could reach 0.993, and the accuracy could reach 0.992. To prevent the occurrence of overfitting, we carried out 10‐fold cross‐validation of six ML algorithms and fivefold cross‐validation of the XGB ML model with the best performance, and the AUC values were all above 0.990. Thus, it showed that the characteristic variables screened by LR could achieve excellent accuracy and clinical application value in the use of the XGB ML model for the prediction of OM in PLC patients.

However, although the ML model was more powerful and accurate than the traditional statistical model, the interpretability of the ML model is relatively more complex, just like the black box. It limits their clinical application and the interpretation of various clinical indicators. For this reason, we explain the characteristic variables and rank the importance of the optimal XGB ML model by using the SHAP library. SHAP is an independent ML model interpretation technique that can explain the black box ML model of global and individual samples and helps explain the relationship between predictors and results in the XGB model.^35^, ^36^ Therefore, based on the optimal ML model, our study aimed to strengthen the global explanation of the application of the XGB model to the prediction of OM, which will help to enhance clinicians' trust in the clinical application of our ML model to provide personalized treatment plans in the process of diagnosis and treatment and technical support for clinical decision‐making. In our study, the ranking of the importance of SHAP ML feature variables was eliminated, and the top three were CA125, ALP, and AFP, respectively. It can be seen that among the various indicators affecting OM in PLC patients, these three indicators made the greatest contribution. This also confirms the conclusions of our previous studies on patients with liver cancer and diabetes or hypertension. The levels of AFP in healthy adults are very low, and some primary tumors will have abnormally high levels of AFP. Therefore, this index can be used to screen for adult tumors, such as liver cancer, ovarian tumors, and other pathological changes.^37^, ^38^ CA125 is also an effective tumor marker that can be used for early diagnosis and monitoring of chemotherapy response in epithelial ovarian cancer, and it is an effective marker for the diagnosis of ovarian cancer.^39^ On the contrary, ALP is a good indicator of liver function in the clinic, and its elevation can be seen in many diseases such as extrahepatic biliary obstruction, liver cancer, and liver cirrhosis. ALP has been proven to be closely related to a variety of metastatic cancers, including metastatic gastric cancer^40^ and metastatic liver cancer,^41^ and can be used as an independent prognostic indicator. The findings of this study are consistent with the above and, thus, show the clinical predictive value of ALP, AFP, and CA125 for OM in PLC patients.

However, there are still some limitations in this study. First, this study was a single‐center retrospective study and lack external validation, and the performance of ML algorithms may vary according to patient characteristics in different regions and the data sets of different institutions. Therefore, in our further research, we would try to obtain multicenter large sample data sets to verify the robustness and repeatability of our model. Second, there were relatively few characteristic variables in our ML algorithm. We would include more clinical indicators in the follow‐up research, carry on the prospective verification of larger sample size, continue to explore the key risk factors of OM, and further modify model parameters to improve the accuracy of the XGB prediction model.

## AUTHOR CONTRIBUTIONS

**Jin‐Qi Sun:** Formal analysis (equal); methodology (equal); resources (equal); validation (equal); writing – original draft (equal); writing – review and editing (equal). **Shi‐Nan Wu:** Formal analysis (equal); methodology (equal); resources (equal); validation (equal); writing – original draft (equal); writing – review and editing (equal). **Zheng‐lin Mou:** Formal analysis (equal); methodology (equal); validation (equal); writing – original draft (equal); writing – review and editing (equal). **Jia‐Yi Wen:** Data curation (equal); formal analysis (equal); methodology (equal). **Hong Wei:** Writing – review and editing (equal). **Jie Zou:** Writing – review and editing (equal). **Qing‐jian Li:** Writing – review and editing (equal). **Zhao‐Lin Liu:** Writing – review and editing (equal). **San‐Hua Xu:** Writing – review and editing (equal). **Min Kang:** Writing – review and editing (equal). **Qian Ling:** Writing – review and editing (equal). **Hui Huang:** Writing – review and editing (equal). **Xu Chen:** Writing – review and editing (equal). **Yi‐xin Wang:** Writing – review and editing (equal). **Xu‐Lin Liao:** Writing – review and editing (equal). **Gang Tan:** Writing – review and editing (equal). **Yi Shao:** Writing – review and editing (equal).

## FUNDING INFORMATION

National Natural Science Foundation (No: 82160195); Jiangxi Province Double Thousand Plan Science and Technology Innovation High‐end Talent Project (2022); Major (Key) R&D Program of Jiangxi Province (No: 2022103; 20181BBG70004; 20203BBG73059); Excellent Talents Development Project of Jiangxi Province (No: 20192BCBL23020).

## CONFLICT OF INTEREST STATEMENT

This study did not receive any industrial support. The authors have no competing interests to declare regarding this study.

## ETHICAL APPROVAL AND CONSENT TO PARTICIPATE

The study methods and protocols were approved by the Medical Ethics Committee of the First Affiliated Hospital of Nanchang University (Nanchang, China) and followed the principles of the Declaration of Helsinki. All subjects were notified of the objectives and content of the study and latent risks and then provided written informed consent to participate.

## DECLARATION OF INTEREST

This study did not receive any industrial support. The authors have no competing interests to declare regarding this study.

## Supporting information


Data S1:

## Data Availability

The datasets used and/or analyzed during the present study are available from the corresponding author upon reasonable request.
