# Supplementary material for: Prediction model of ocular metastasis from primary liver cancer: Machine learning‐based development and interpretation study
Source: Cancer Med. 2023 Oct 5;12(20):20482–96. doi: 10.1002/cam4.6540 (PMC10652349; doi:10.1002/cam4.6540)
Supplement: Supplementary file 1 — Data S1: [file CAM4-12-20482-s001.docx]

**Supplementary Materials**

Readers can also use these detailed parameter settings to reproduce our model with *Python software*. The *model_parameter_settings.txt* can also be downloaded from the following publicly available github repositories.

(https://github.com/Wu-Shi-Nan/liver_cancer_ml_parameters/blob/main/model_parameter_settings.txt )
